# Case report: apatinib plus selexipag as a novel therapy for pulmonary tumor thrombotic microangiopathy accompanied by pulmonary hypertension associated with gastric carcinoma

**DOI:** 10.1097/MD.0000000000029412

**Published:** 2022-07-15

**Authors:** Guofeng Ma, Dan Wang, Xiaoling Xu, Li Liang, Li Xu

**Affiliations:** a Regional medical center for National institute of respiratory diseases, Sir Run Run Shaw Hospital, Zhejiang University School of Medicine, Hangzhou, China; b Department of Radiology, Sir Run Run Shaw Hospital, Zhejiang University School of Medicine, Hangzhou, China.

**Keywords:** oral prostacyclin receptor agonist, PTTM, pulmonary hypertension, VEGFR antagonist

## Abstract

**Rationale:**

PTTM is a rare but fatal disease, characterized by endothelial intimal proliferation and pulmonary hypertension due to micro-vascular remodeling. In view of the poor prognosis, new effective strategies are urgently required.

**Patient concerns and diagnosis:**

A 51-year-old woman was admitted to hospital for acute progressive dyspnea and dry cough. Clinical tests revealed hypercoagulable state and signs of severe pulmonary hypertension, without evidence of pulmonary embolism on contrast-enhanced CT. CT showed interlobular septal thickening and diffuse ground-glass opacity. Lung perfusion scan indicated multiple segment defect. Further right heart catherization proved a significant increase in pulmonary vascular resistance.

**Interventions:**

A combination therapy of apatinib and selexipag was administered for treatment of PTTM. The conventional therapies of ventilation, anticoagulation and diuretic medicines were initiated after admission.

**Outcomes:**

Symptoms of PTTM were ameliorated with a reduction in pulmonary artery pressure. The resolution of interlobular septal thickening and ground-glass opacity on CT constituted the clinical benefits from treatment.

**Lessons:**

Patient with PTTM will benefit from the combination strategy of apatinib, a VEGF-receptor antagonist, and selexipag, an oral prostacyclin receptor agonist.

## 1. Introduction

Pulmonary tumor thrombotic microangiopathy (PTTM) is a rare but lethal disease characterized by acute onset progressive dyspnea and severe pulmonary hypertension in carcinoma patients.^[1]^ Endothelial cell proliferation and micro-vasculature remodeling are the main pathophysiologic mechanisms. Several cytokines including VEGF and PDGF have been demonstrated as the key components in the treatment of PTTM.^[2,3]^ In the light of the extremely poor prognosis despite use of imatinib through the PDGF inhibition pathway, an effective treatment strategy is urgently needed. In this case study, a novel combination therapy of apatinib plus selexipag for PTTM is introduced.

## 2. Case Presentation

A 51-year-old woman without relevant past history was admitted to our hospital for progressive dyspnea and dry cough for 1 week. Her dyspnea worsened when on exertion, and she felt breathless after a walk for 100 m with WHO functional class III symptoms. She showed no symptoms of hemoptysis, chest pain or fever. Oral cough medicines failed to relieve her symptoms. Subsequent heart ultrasonography outside the hospital indicated severe pulmonary hypertension, but there were no positive findings from CT pulmonary angiography.

Vital signs at admission are summarized as follows: temperature 36.7°C, blood pressure 102/60 mm Hg, respiratory rate 19 bpm, pulse rate 67 bpm, and oxygen saturation ranging from 94%–97% on room air. Physical examination was nonspecific except for bilateral light rales. No distended jugular vein was seen, and no noticeable abnormal sounds were auscultated on heart and lung examinations.

Laboratory data are given as follows (Table 1): arterial blood gas analysis showed hypoxemia with partial pressure of oxygen, 66 mm Hg; oxygen saturation of 94% upon room air inhalation. Blood tests revealed a positive result for D-dimer 1.57 (0–0.5 µg/mL) and lactase 2.2 (0.9–1.7 mmol/L). Tumor marker levels were significantly elevated: ca199 142 (<37 IU/mL), ca211 11.7 (0–3.3 µg/L) and ca724 28.96 (0–6.9 U/mL). Thyroid function, auto-immune antibodies and AIDS antibodies were tested negative. Thrombosis screening including protein S, protein C, antithrombin, antiphospholipid antibody series, lupus anticoagulant and homocysteine showed no positive results. There were no additional hematologic and biochemical abnormalities.

**Table 1:** Laboratory results on admission.

Hematology		Biochemistry	
WBC	5.3*10^9^/L	TP	69.6 g/L
Hb	130 g/L	Alb	37.9 g/L
Plt	171*10^9^/L	ALT	16 U/L
Neutro	82.4%	AST	24 U/L
Lymph	10.3%	LDH	226 IU/L
Coagulation		T-bil	6.5 µmol/L
APTT	34.9 s	Cre	63 µmol/l
PT	12.7 s	CK	58 U/L
INR	0.98	Troplin I	<0.01 ng/ml
D-dimer	1.57 µg/ml	NT-proBNP	245 pg/ml
Fibrinogen	3.93 g/L	CRP	3.2 mg/L
Blood gas analysis (room air)			
PH	7.416		
PaO_2_	66.3 mm Hg		
PaCO_2_	41.7 mm Hg		
P/F	316		
LAC	2.2 mmol/L		

**Figure 1.**
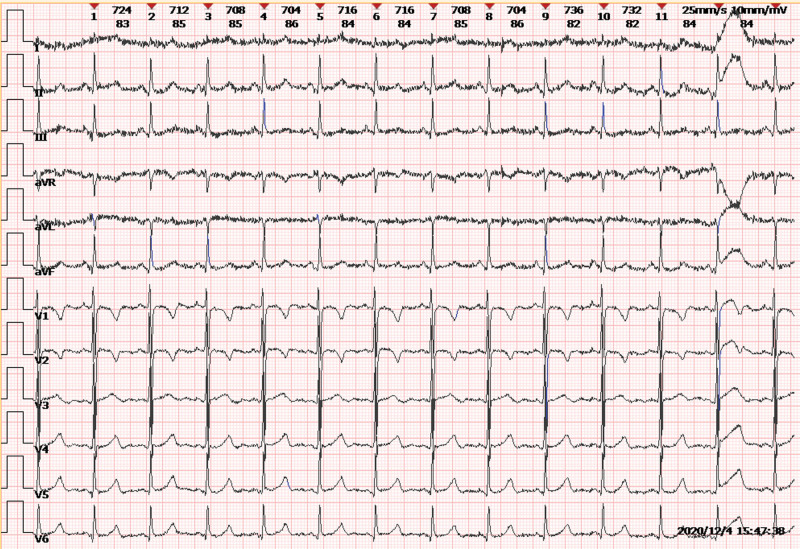
T wave inversion in right V1-2 leads.

Electrocardiogram (ECG) exhibited normal sinus rhythm and T wave inversion in right V1-2 leads (Fig. 1). Transthoracic echocardiography revealed severe pulmonary hypertension with estimated right ventricular systolic pressure of 111 mm Hg, accompanied by dilated right ventricle, paradoxical movement of the interventricular septum and moderate to severe tricuspid regurgitation, but preserved left ventricular systolic function with an ejection fraction of 69.7%. Detailed parameters are presented in Table 2.

**Table 2:** Echocardiogram parameters.

Parameters	Result (2021.12.4)	Result (2021.12.21)	Reference value
EF	69.7%	60%	>50%
AAO	33.5		28–38 mm
AO	18		20–35 mm
LA	50.6*30.6*27		19–35 mm
RA	52.2*33.6	52*38.8	33–41 mm
PA	31	26	12–26 mm
LPA	13		14–19 mm
RPA	20.6		15–21 mm
RVD	47.8	39.6	7–23 mm
TV	5.06	3.3	0.3–0.7 m/s
IVC	13.2		19–21 mm
RVSP	111	49	15–30 mm Hg

**Figure 2.**
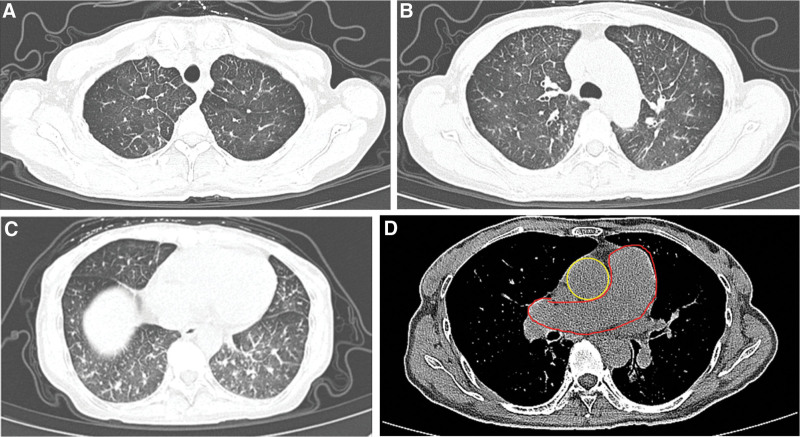
Ground-glass opacity, interlobular septal thickening and enlarged pulmonary artery in computed tomography.

High-resolution CT showed mosaic pattern with ground-glass opacity and interlobular septal thickening. Mediastinal window suggested the main pulmonary artery was enlarged (Fig. 2). Lung ventilation and perfusion scan showed mismatched perfusion defects in the right middle internal segment, indicating possible pulmonary embolism.

**Figure 3.**
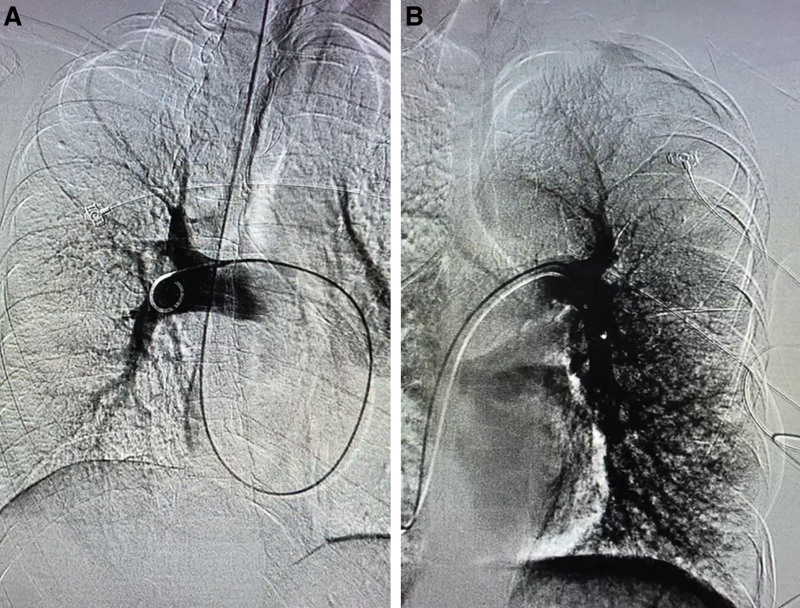
Pulmonary artery filling defect in pulmonary angiography.

Due to the possibility of pulmonary thromboembolism, pulmonary angiography was performed. Results showed distal branches of the pulmonary artery had become sparse and thin (Fig. 3). Further examination of right heart catheterization revealed pulmonary hypertension with pulmonary artery pressure of 43/14 (25) mm Hg, and the pulmonary arterial wedge pressure was 3 mmHg (Table 3). The serum VEGF level was tested as 169 ng/L, significantly higher than that in healthy individuals. The aspiration cytology obtained from the pulmonary artery via right heart catheterization showed a negative result.

**Table 3:** Right heart catheterization.

	Systolic pressure (mm Hg)	Diastolic pressure (mm Hg)	Mean pressure (mm Hg)	PaO_2_ (mm Hg)	SaO_2_ (%)
BP	105	63	77		
CVP			13	43.9	77.7
RA	7	1	3	40.3	76.5
RV	41	0	18	47.3	83.8
PA	43	14	26	44.1	84.9
HR	69 bpm	PAWP	3 mmHg	PVR	596dyn.s.cm^−5^

Selexipag (0.2 mg/day, BID), an oral prostacyclin receptor agonist, was administered to decrease her pulmonary pressure. Furosemide and spironolactone were given to reduce cardiac load. Enoxaparin (0.4 mL/day, QD) was prescribed as a preventive anticoagulation therapy. Electrocardiogram was performed to closely monitor her response to treatment, and ventilation was made by mask. After initial 5-day treatment, the patient’s symptoms of breathlessness and cough were partially relieved. Right ventricular systolic pressure had decreased from 111 to 58 mm Hg. Due to the unknown reason for the patient’s pulmonary hypertension and significantly elevated tumor indicators, PET-CT was administrated to determine causative factors for occult spread.

**Figure 4.**
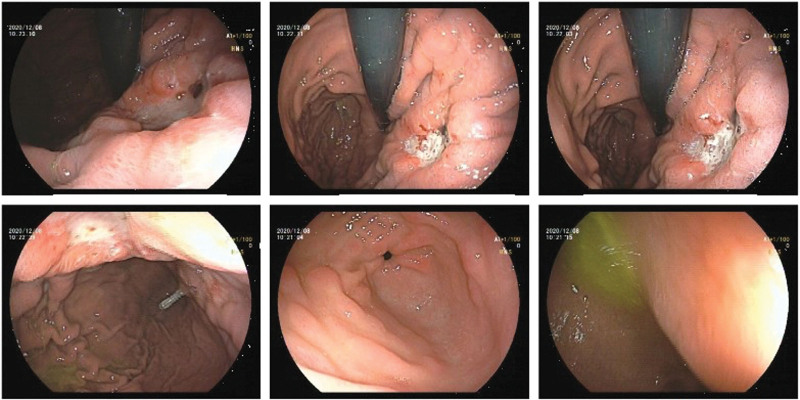
Gastroscopy picture.

Results clearly indicated the curvature of the stomach wall was reduced and thickened; multiple bone deterioration was observed in cervical, thoracic, lumbar spine and pelvis regions. All these parts appeared with increasing FDG metabolism uptake, which indicated a possibility of malignancy and multiple metastases. Gastroscopy was performed, and irregular gastric ulcer was seen. Further biopsy and pathology analysis demonstrated poorly differentiated gastric adenocarcinoma (Fig. 4).

**Figure 5.**
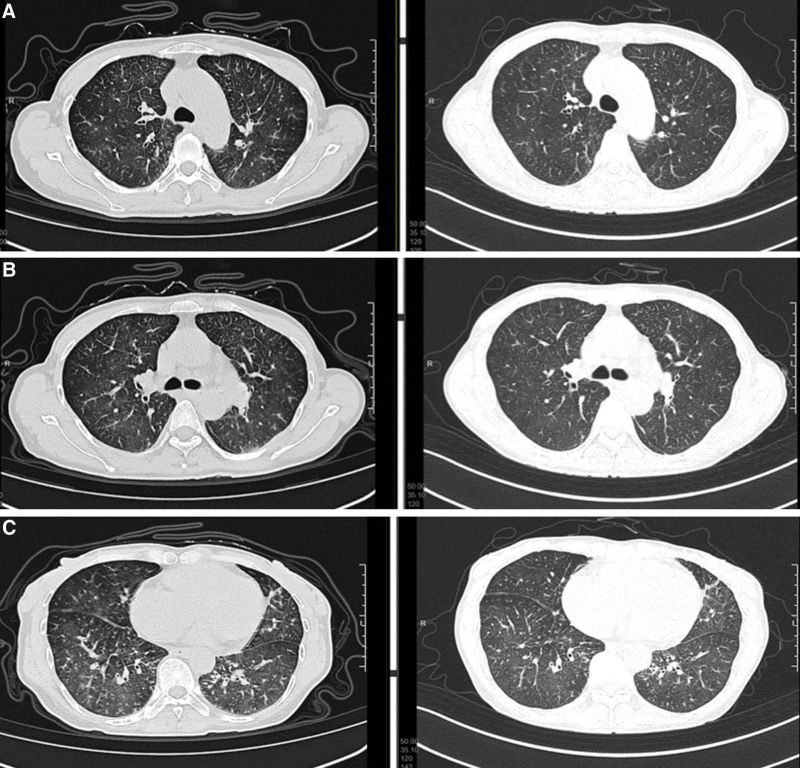
Comparation of computed tomography on day 1 and day 19.

According to the presenting symptoms and characteristic laboratory and imaging findings, as well as the biopsy results of advanced stomach cancer, the diagnosis of pulmonary tumor thrombotic microangiopathy was considered. The high VEGF index of 169 verified the diagnosis. On Day 5 after admission, tegafur was initiated for the treatment of advanced gastric cancer. The patient experienced an intolerable side effect of fatigue. Tegafur was discontinued and replaced with apatinib (250 mg/day, QD) for antitumor treatment on Day 13 after admission. As the apatinib therapy was off-label, a written informed consent was obtained from the patient. She subsequently reported a much more comfortable feeling with recovery of activity endurance and improvement of physical state. She was then able to move about the ward independently. The symptoms of dyspnea and cough had also been relieved. CT was conducted to assess the therapeutic effects on Day 19 after admission. The CT image showed the absorption of ground-glass exudation lesions in the lower zone of bilateral lung (Fig. 5). Further examination with echocardiogram showed that RVSP gradually decreased from 111 to 49 mm Hg (Table 2).

Apatinib (250 mg/day), selexipag (0.2 mg every 12 hours) and enoxaparin (0.4 mL/day) were prescribed at discharge. Outpatient ECHO was performed at 2-week follow-up evaluation. Right ventricular systolic pressure improved from 49 to 26 mm Hg. Due to financial burden and misunderstanding of the need for continuous medication, she suspended all medications. Unfortunately, she died due to suddenly worsened respiratory symptoms.

## 3. Discussion

Firstly proposed by Von Herbay et al in 1990,^[1,4]^ pulmonary tumor thrombotic microangiopathy (PTTM) is a rare but fatal disease characterized by rapidly progressive hypoxia and severe pulmonary hypertension in patients with carcinoma with occult spread. It can be challenging to make a definite diagnosis before antemortem status.^[5]^ Its acute onset symptoms of dyspnea accompanied by pulmonary hypertension often lead to a misdiagnosis of pulmonary thromboembolism. PTTM shows negative results rather than positive findings of occlusion in the pulmonary artery as revealed by high-resolution CT.^[6]^ However, PTTM is much more serious than pulmonary embolism due to characteristic rapid worsening and short survival period.^[1,2,7]^

Based on the existing research evidence, the incidence of PTTM ranges from 0.9% to 3.3% in patients with carcinoma as proved by autopsy postmortem.^[1,2,8]^ However, the incidence increases to 16.7% in the cases with gastric cancer.^[9]^ Von Herbay et al reported 11 of 21 patients who were firstly considered PTTM through autopsy were finally diagnosed with gastric cancer.^[1]^ Other research also found high prevalence of PTTM in patients with stomach cancer.^[5,9,10]^ In contrast, PTTM is relatively less frequently associated with other kinds of cancer.^[1,2,5]^ PTTM occurs not only at advanced carcinoma stages, but also at early stages. The most prevalent histological subtype is adenocarcinoma rather than adeno-squamous, signet-ring or poorly differentiated subtype.^[2,11]^ As a fatal complication, less attention had paid been to this syndrome.^[7]^ When the diagnosis of PTTM is considered, an active screening strategy should be adopted in searching for occult malignancy.

The pathophysiology of PTTM is still ambiguous.^[5,12]^ As some researchers have postulated, 1 possible internal mechanism of PTTM pathogenesis initiates with disseminated circulating tumor cell attachments and damage to the endothelial cells of the pulmonary micro-vasculature, which in turn switches on a coagulation cascade causing platelet aggregation and secretion of various cytokines promoting diffuse fibromuscular intimal proliferation. Remodeling of pulmonary vasculature characterized by luminal stenosis or obstruction leads to an increase in vascular resistance and results in pulmonary hypertension.^[3,4,11,12]^ Postmortem autopsy or biopsy antemortem results have revealed the morphologic findings of PTTM are similar to microscopic tumor embolization mainly characterized by mechanical occlusion of pulmonary vessels, whereas intimal proliferation is rare in the latter situations.^[1,2,13]^

Several cytokines and molecules secreted by carcinoma cells, including vascular endothelial growth factor (VEGF),^[6,9,10]^ platelet-derived growth factor PDGF^[5,10]^, tissue factor (TF)^[14]^ and osteopontin,^[15–17]^ play key roles in the complex mechanism of PTTM. Uruga H et al (2013) found a high expression rate for these molecules in carcinoma patients diagnosed with PTTM, as the immunoreactive rates of VEGF, PDGF, TF, and osteopontin were 96.6%, 62%, 100%, and 62%, respectively.^[2]^

Angiogenesis is a normal physiological process occurring in daily life, which is crucial for wound healing, growth, and healthy organ function. However, aberrant angiogenesis also happens in a wide range of disorders such as cancer and coronary atherosclerosis. The balance between endogenous proangiogenic and antiangiogenic is critical for maintaining healthy.^[18,19]^ Since tumor progression and metastasis are angiogenesis-dependent processes, antiangiogenesis therapy is a promising method for cancer treatment. As VEGF signaling is a main channel to promote angiogenesis, targeted therapeutic strategies on the inhibition of VEGF or VEGF-receptor^[3]^ signaling systems are promising.^[20–22]^ Many studies have shown the important role of VEGF in the regulation of endothelial cell proliferation and vascular remodeling in patients with PTTM.^[23–25]^ PDGF released by tumor cells is a stimulator to initiate macrophage recruitment and upregulate VEGF expression, which will induce intimal proliferation, promote smooth muscle cell proliferation and migration.^[15,17]^ TF, released by tumor cells and endothelial cells, is responsible for initiation of coagulation cascade and formation of fibrin clots.^[16,17]^ Osteopontin arising from endothelial intima in the presence of inflammation participates in vascular remodeling.^[16,17]^

The symptoms of PTTM tend to be nonspecific. Patients presents with cough, dyspnea and other signs of hypoxia in rapid clinical course, misleading diagnosticians to other diagnosis results.^[18]^

Blood test results show increased D-dimer and FDPs which originate from activation of the coagulation pathway and fibrin degradation. Other characteristics include anemia, thrombocytopenia and disseminated intravascular coagulation.^[5]^ Refractory right-side heart failure induces elevated parameters of myocardial enzymes, cardiac troponin and type B natriuretic peptide.^[18]^ The results of blood gas analysis will come out with type 1 respiratory failure accompanied by low alveolar oxygen partial pressure and blood oxygen saturation. Elevated tumor indicators indicate carcinoma or occult tumors.^[3,9]^

Doppler echocardiogram shows the signs of pulmonary hypertension with elevated average right ventricular systolic pressure and right ventricular enlargement or dysfunction.^[5]^ Radiological examination of PTTM always appears with various nonspecific characteristics as CT findings including bilateral lung ground-glass opacification indicating septal thickening of pulmonary alveolar cells, scattered distribution of centrilobular nodules, and tree-in-bud sign images. In mediastinal window, an enlarged pulmonary artery can be seen.^[19–21]^ Radionucleotide ventilation perfusion scans show multiple small segments or subsegment mismatched filling defect.^[2,22]^ 18F-FDG-PET scan presents with local uptake areas, suggesting occult carcinoma possibility and metastasis region.^[2]^

For the limited presenting symptoms of PTTM, it is difficult to establish an antemortem diagnosis based on preliminary examinations. Diagnosticians should consider PTTM when patients appear with new-onset pulmonary hypertension accompanied by the symptoms of dyspnea or cough, but lack evidence of pulmonary artery embolism by contrast-enhanced CT.^[1,3]^

Biopsy specimens from patients with PTTM strongly contribute to a definitive diagnosis. However, it is difficult to access enough biopsy specimens antemortem.^[1,2,9]^ Right heart catheterization is a new alternative method to diagnose PTTM antemortem. A Swan-Ganz catheter embedded into the pulmonary artery can be used to collect blood samples for cytological analysis of malignant tumors.^[7,23–25]^ Likewise, transbronchial needle aspiration of lung and lymph node specimens have been performed for pathology analysis with minimally invasive procedures.^[26,27]^

In this case, right heart catheterization was tried to collect blood samples from the pulmonary artery to detect circulating tumor cells. Unfortunately, no malignant cells were found. Given the rapid deterioration process of her physical condition, pathological diagnosis based on biopsy seemed challenging. Without searching for a more aggressive diagnostic strategy due to the rapid progressive clinical course, a clinical diagnosis of PTTM was made based on the combination analysis of clinical characters and corresponding examination results, (1) acute onset of symptoms with a rapidly progressive course, (2) CT and echocardiogram which showed the signs of pulmonary hypertension and then further confirmation with right heart catheterization. Ventilation perfusion showed multiple subsegment defects without positive evidence on contrast-enhanced CT, (3) hypercoagulative state with elevated D-dimer, (4) high VEGF expression, and (5) definite diagnosis of advanced gastric cancer. Given the possible diagnosis, conventional therapies of ventilation, anticoagulation, and diuretic medicines were initiated after admission.

In view of the relatively complex pathophysiology of PTTM involving interplay between many types of cells and various cytokines including PDGF and VEGF, many researches targeting PDGF for treatment of PTTM had been carried out with great interest. As it is a key component for intimal proliferation inducing vascular stenosis and pulmonary hypertension. PDGF was found by way of overexpression in smooth muscle and endothelial cells in patients with idiopathic pulmonary hypertension. Imatinib, a PDGF receptor tyrosine kinase inhibitor, was historically known as an antiproliferative agent for chronic myeloid leukemia and has been proven to be effective in inhibiting proliferation of pulmonary artery smooth muscle cells and vascular remodeling.^[5,22,26,28]^ Ogawa et al (2013) reported a good response in patients with PTTM to the treatment with imatinib in reduction of pulmonary artery pressure and circulation stabilization.^[29]^ PDGF was correspondingly reduced after the initiation of imatinib treatment.^[29,30]^ Numerous follow-up studies confirm that patients diagnosed with PTTM benefit from prompt initiation of imatinib treatment, which prolongs the survival period.^[22,29,31]^ However, imatinib alone has not been found to be of great therapeutic benefit, and is not effective enough in the treatment of PTTM in the rapid progressive process. In addition to imatinib, bevacizumab, as a VEGF inhibitor, has also been found slightly effective in treatment of PTTM with pulmonary hypertension.^[7]^ Other research has shown the limited therapeutic benefit in thrombolysis and traditional chemotherapy.^[32–34]^ ECMO is also considered as a supplemental therapy for bridging of critical ill patients.^[35]^

The prognosis of PTTM is extremely poor as patients will exhibit irreversible pathologic changes of pulmonary microvascular and arterial intimal thickening and fibrotic proliferation. Therefore, a timely targeted therapy to curb the pathophysiology of pulmonary vessel remodeling is critically important.

Since PDGF inhibitors such as imatinib have limited therapeutic effects in critically ill patients, more effective approaches are urgently needed. As a result, this novel approach using apatinib plus selexipag for treatment of PTTM was conducted.

Selexipag is a novel oral prostacyclin receptor agonist with proven clinical efficacy and good tolerability in the treatment of pulmonary hypertension. Prostacyclin secreted by endothelial cells causes vasodilation, prevents platelet aggregation, and inhibits smooth muscular cell proliferation. Activation of the prostacyclin pathway is a significant method to decrease pulmonary vascular pressure.^[36–38]^ In the GRIPHON trial, selexipag was found to significantly reduce the risk of primary composite endpoint compared to placebo.^[39]^ As PTTM patients tend to appear with hypercoagulation state inducing fibrotic clot formation, endothelial cells and smooth cell proliferation leading to luminal stenosis, the administration of selexipag may block coagulation cascade and relieve the intimal proliferation process.

Over the past many years, VEGF had been verified as an attractive therapy for inhibition of angiogenesis. VEGF binds to VEGFR, activating the downstream signaling pathways which promotes the process of endothelial cells proliferation, migration and neovascularization.^[40–42]^ It has been demonstrated that angiogenesis is a main prerequisite factor and facilitator for tumor growth and metastasis.^[43]^ In the light of these mechanisms, medicines and other therapeutic strategies targeting VEGF pathways to block normal angiogenesis have proven to be reasonably effective treatments for advanced cancers. VEGF is an important molecule playing a crucial role in the pathophysiologic mechanism of PTTM. The level of VEGF was found to be higher in patients with PTTM. Apatinib, as a novel and highly selective tyrosine kinase inhibitor targeting VEGF receptor-2, has shown great effects as an antiangiogenic therapy through down-regulation of downstream signaling pathways. It plays a powerful role in the inhibition of tumorigenesis, blocking tumor growth, and metastasis.^[24,44]^ It had been proven effective in promoting apoptosis and autophagy in gastric cancer cells and was suggested as a third-line therapy for intractable and advanced gastric carcinomas.^[45,46]^

Given patient consent for use of off-label medications and tentative, at least short-term success in this preliminary study, further research on combination treatment of apatinib and selexipag appears to be warranted in treatment of PTTM. This therapy ameliorated symptoms of PTTM with a reduction in pulmonary arterial pressure and substantial improvement in physical activity endurance. The resolution of interlobular septal thickening and ground-glass opacity as revealed by CT were other clues to suggest clinical benefits from this treatment.

This was a successful case for PTTM patient treated with the combination therapy of apatinib and selexipag, despite final poor prognosis owing to unauthorized withdrawal. As this was a new and reasonably successful attempt at treatment with VEGFR antagonist plus novel oral drug targeting pulmonary hypertension, ongoing research into similar cases should be carried out to verify the efficacy of this therapy.

## 4. Conclusions

Given the rapid progressive course of PTTM and lethality of this disease, we should increase the awareness of PTTM, which is presented as new-onset pulmonary hypertension, associated with previously or newly diagnosed tumors for an early diagnosis. The combination therapy of apatinib plus selexipag may bring new hope for patients with PTTM.

## Author contributions

GM wrote the case report, LX helped revise the manuscript, DW helped choose the images, and XX, LL treated this patient. All authors read and approved the final manuscript.
